# First Application of 3,5-Dinitrocatechol and Benzalkonium Chloride in Cloud Point Extraction: Spectrophotometric Determination of Vanadium(V)

**DOI:** 10.3390/molecules31193364

**Published:** 2026-09-22

**Authors:** Petya Racheva, Antoaneta Saravanska, Andrea Gajdošová, Kirila Stojnova, Vassil Delchev, Jana Šandrejová, Kiril Gavazov

**Affiliations:** 1 Department of Chemical Sciences, Faculty of Pharmacy, Medical University of Plovdiv, 120 Buxton Bros Str., 4004 Plovdiv, Bulgaria; petya.racheva@mu-plovdiv.bg (P.R.); antoaneta.saravanska@mu-plovdiv.bg (A.S.); 2 Department of Analytical Chemistry, Institute of Chemistry, Faculty of Science, Pavol Jozef Šafárik University in Košice, Moyzesova 11, 04154 Kosice, Slovakia; andrea.gajdosova@student.upjs.sk (A.G.); jana.sandrejova@upjs.sk (J.Š.); 3 Department of General and Inorganic Chemistry with Methodology of Chemistry Education, Faculty of Chemistry, University of Plovdiv ‘Paisii Hilendarski’, 24 Tsar Assen St., 4000 Plovdiv, Bulgaria; kirila_stoinova@abv.bg; 4 Department of Physical Chemistry, Faculty of Chemistry, University of Plovdiv ‘Paisii Hilendarski’, 24 Tsar Assen St., 4000 Plovdiv, Bulgaria; vdelchev@uni-plovdiv.net

**Keywords:** vanadium, spectrophotometric determination, 3,5-dinitrobenzene-1,2-diol, ternary complex, centrifuge-less cloud point extraction, mixed micellar system

## Abstract

The ability of 3,5-dinitrocatechol (3,5-DNC) to form anionic species with vanadium(V) in a mildly acidic medium was exploited to develop a spectrophotometric method for vanadium determination. The method is grounded on the formation of an ion-association complex with benzalkonium chloride (BAC), which is efficiently extracted into a surfactant-rich phase formed in a mixed micellar medium consisting of Triton X-114 and BAC. The stoichiometry of the extracted complex was established as (BA^+^)[VO(3,5-DNC)_2_], and the formula of its anionic component, [VO(3,5-DNC)_2_]^−^, was verified by quantum-chemical calculations at the B3LYP/6-311G level of theory and by comparison of the experimental absorption spectrum with simulated theoretical spectra. The results indicate that the thermodynamically more stable anti isomer is preferentially formed and extracted. The developed novel centrifuge-less cloud point extraction–spectrophotometric method exhibits an absorption maximum at 426 nm, an apparent molar absorptivity of 1.6 × 10^6^ L mol^−1^ cm^−1^, a limit of detection of 1.7 ng mL^−1^, and a linear range of 5.7–29.1 ng mL^−1^. The method was successfully applied to the determination of vanadium in spent catalysts and vanadium-based dietary supplements. Its analytical performance and sustainability were evaluated using modern assessment tools, including RGBfast.

## 1. Introduction

Vanadium is classified as a dispersed trace element in the Earth’s crust and, according to the RRUFF Project database, occurs in approximately 295 mineral species [1]. Recognized as a critical raw material by the European Union [2], vanadium enters the biosphere through both natural and anthropogenic pathways. Owing to its strategic and economic importance [3], vanadium has re-emerged as an element of environmental concern, driven by evolving industrial practices and the generation of novel waste streams [4,5].

The predominant vanadium anthropogenic oxidation state, V(V), is generally considered to be more mobile and hazardous than V(IV) and V(III). Due to its chemical similarity to phosphate, this form can disrupt essential biochemical pathways and competitively block key physiological processes [6]. Conversely, low concentrations of vanadium may exert beneficial biological effects, including potential anticancer activity through a wide range of mechanisms of action [7,8,9] and improvements in glucose metabolism in individuals with type 2 diabetes [10,11]. Vanadium-containing dietary supplements have been promoted for glycemic control, enhancement of athletic performance, and stimulation of muscle growth. However, a major challenge in the development and application of vanadium-based drugs and dietary supplements is achieving an optimal balance between therapeutic efficacy and toxicity, a problem that remains unresolved despite extensive research efforts [11,12,13].

There are numerous methods available for determining V(V). Spectrophotometric methods are widely used because they are simple, accessible, quick, and less expensive than other instrumental methods [14,15,16,17,18]. Combining them with preconcentration procedures can significantly improve analytical performance, particularly in terms of sensitivity and selectivity, thereby expanding their scope of application considerably.

3,5-Dinitrocatechol (3,5-DNC; Figure 1a) is a commercially available reagent included in the IUPAC list of essential spectrophotometric reagents [19]. It is also known for its biological activity under the designation OR-486 and is considered the progenitor of a class of compounds used in medicine as catechol-O-methyltransferase (COMT) inhibitors [20,21]. In combination with basic dyes, 3,5-DNC has been successfully applied to the spectrophotometric determination of post-transition, inner-transition, and transition elements, including vanadium [22,23]. However, the reported highly sensitive procedures rely on conventional preconcentration techniques, such as liquid–liquid extraction (LLE) [24] and solvent flotation [25], which require substantial amounts of toxic organic solvents. This drawback compromises one of the key advantages of 3,5-DNC, namely its favorable environmental and safety profile. According to its Safety Data Sheet (SDS), 3,5-DNC is not considered a hazardous substance and therefore does not require hazard statements, hazard codes, or hazard pictograms on its label [26].

The objective of this study was to develop a 3,5-DNC-based spectrophotometric method for the determination of vanadium(V) using cloud point extraction (CPE) as a preconcentration approach. CPE is a green separation and preconcentration technique that employs surfactants instead of toxic organic solvents [27,28,29,30,31,32]. It is increasingly being adopted as an alternative to conventional extraction techniques, often using the same reagents after appropriate optimization, although the composition of the extracted hydrophobic species may differ from that obtained by conventional extraction methods [33]. 

A distinctive feature of the procedure proposed in this work is the use of a mixed-micellar medium (MM) composed of Triton X-114 (TX114) and benzalkonium chloride (BAC; Figure 1b). In this extraction system, BAC performs a dual function, acting both as a cationic surfactant and as a chain-type ion-association reagent [34].

Another feature of the developed procedure is its operational simplicity, which stems from the elimination of salting-out agents, often employed to improve extraction efficiency, and the implementation of centrifuge-less cloud point extraction (CL-CPE), thereby further reducing equipment requirements.

It should be emphasized that neither 3,5-DNC nor BAC has previously been employed in CPE procedures for metal preconcentration. To the best of our knowledge, among catechol derivatives, only 4-nitrocatechol [35,36,37] and pyrogallol [38,39,40] have been used for this purpose in combination with cationic ion-association reagents, such as Aliquat 336, cetylpyridinium chloride, and Safranin T.

## 2. Results and Discussion

### 2.1. CPE–Spectrophotometric Optimization

#### 2.1.1. Absorption Spectrum

Two research groups have investigated vanadium(V)-3,5-DNC complexes. The formation of binary complexes in aqueous solution with absorption maxima (*λ*_max_) at 419–428 nm [25,41] has been reported, as well as ion-association complexes with Brilliant Green [24] (λ_max_ = 630 nm) and Rhodamine B [25] (λ_max_ = 555 nm) in nonaqueous media.

Figure 2 presents the absorption spectrum of the V(V)–3,5-DNC–BAC complex extracted into the surfactant-rich phase (SRP). The maximum is located at 426 nm, which is essentially identical to that of the anionic V(V)–3,5-DNC species in aqueous solution. This finding is readily rationalized by the fact that, unlike Brilliant Green and Rhodamine B, which possess intrinsic absorption bands in the visible region, the BAC cation (BA^+^) involved in the present ion-association complex is colorless.

#### 2.1.2. Optimum Acidity

Sulfuric acid was used to establish optimum acidity, as suggested by previous studies [24,42]. Owing to its non-volatile nature, this acid does not cause complications in CPE. The effect of H_2_SO_4_ concentration on the extraction process is shown in Figure 3. Maximum absorbance was observed at *c*(H_2_SO_4_) in the range of (8–9) × 10^−3^ M, corresponding to a pH of approximately 2.0–2.1. Further studies were conducted at *c*(H_2_SO_4_) = 8.9 × 10^−3^ M.

#### 2.1.3. Optimum Reagent Concentrations

The effects of 3,5-DNC and BAC concentrations on the extraction process are shown in Figure 4 and Figure 5, respectively. Saturation was achieved at a relatively low concentration of 3,5-DNC (1 × 10^−4^ M), whereas the optimum concentration of BAC was approximately nine times higher. Accordingly, all subsequent experiments were performed at *c*(3,5-DNC) = 1 × 10^−4^ M and *c*(BAC) = 9.3 × 10^−4^ M.

#### 2.1.4. Effect of TX114 Mass Fraction

The effect of the TX114 mass fraction, *w*(TX114), on the extraction efficiency is shown in Figure 6. Maximum extraction was achieved at *w*(TX114) values of 0.86–0.93%. Therefore, a TX114 mass fraction of 0.90% was selected as the optimum value.

#### 2.1.5. Effect of Incubation Temperature and Time

The effect of incubation temperature on the extraction process was evaluated over the range of 50–70 °C (Figure 7). Maximum absorbance was achieved at temperatures of 65 °C and above. The minimum incubation time required to complete the CL-CPE process at 65 °C was 60 min (Figure 8).

Preliminary experiments involving ultrasound-assisted CPE, conducted using the equipment described in [40], showed no improvement in the extraction efficiency and were therefore not investigated further.

#### 2.1.6. SRP Treatment

After incubation in the water bath, the samples were briefly cooled under running water. They were then chilled in a refrigerator at −20 °C for 25 min, during which the viscosity of the SRP increased sufficiently to allow easy phase separation by decantation of the upper aqueous phase. Prior to spectrophotometric measurement, the viscosity of the separated SRP was reduced by dilution with ethanol (300 μL) and water to a final volume of 2.0 mL.

Table 1 summarizes the optimized experimental parameters. Under the selected conditions, the absorbance remained stable for minimum two hours.

### 2.2. Formula, Chemical Equations, and Isomerism

#### 2.2.1. Stoichiometric Ratios, Complex Formation Equation, and Conditional Extraction Constant

The molar ratios in the ternary complex were determined using the straight-line method [43] (Figure 9) and the mobile equilibrium method [44] (Figure 10). Both methods yielded identical results, namely 3,5-DNC:V(V) = 2:1 and BAC:V(V) = 1:1. These findings indicate that the formation of the extracted complex can be represented by Equation 1, which is consistent with the predominant forms of V(V) [45,46] and 3,5-DNC (denoted as H_2_L) [47] under the experimental conditions employed.

2H_2_L_(aq)_ + VO_2_^+^_(aq)_ + BA^+^_(aq)_ = (BA^+^)[VO(L)_2_]_(SRP)_ + 2H^+^_(aq)_ + H_2_O (1)

The conditional extraction constant (*K*_ex_), corresponding to Equation (1), was evaluated using the Holme–Langmyhr method [48] based on five experimental points. The calculated value of log *K*_ex_ was 4.2 ± 0.3 (mean ± standard deviation, SD). A statistically indistinguishable value was obtained using the mobile equilibrium method: log *K*_ex_ = 3.9 ± 0.5 [44] (Figure 10, straight line 2).

#### 2.2.2. Ground-State Equilibrium Geometries, Isomerism, and Theoretical Spectra

The proposed formula of the extracted complex was verified by theoretical calculations performed at the B3LYP/6-311G level of theory and by comparison of the experimental and theoretical absorption spectra. Since benzalkonium cations do not absorb in the visible region, only the anionic species, [VO(L)_2_]^−^, was included in the calculations. This approach has previously been shown to provide a satisfactory agreement between theoretical and experimental spectra for ion-association complexes containing colorless cations [49,50].

Owing to the asymmetry of the ligand molecule (3,5-DNC), two geometric isomers of the anionic complex, syn and anti, are possible, differing in the relative orientation of the NO_2_ groups (Figure 11). In both isomers, the vanadium center adopts a slightly distorted square-pyramidal coordination geometry, with only minor differences in the V–O bond lengths.

Based on the optimized structures, vertical excitation energies were calculated using time-dependent density functional theory (TD-DFT) to simulate the theoretical UV–Vis absorption spectra. Comparison of the calculated and experimental spectra (Figure 12) indicates that Structure 2 is in better agreement with the experimental spectrum, providing stronger support for its assignment as the more likely structure of the extracted complex. This assignment is further supported by the calculated thermodynamic parameters, which indicate that Structure 2 is more stable than Structure 1. The electronic energy of Structure 2 is approximately 2.6 kJ mol^−1^ lower than that of Structure 1, while the Gibbs free energy is approximately 4.0 kJ mol^−1^ lower. Thus, both the spectral comparison and the thermodynamic calculations consistently favor Structure 2. Although these energy differences are relatively small, their agreement with the spectroscopic results provides additional support for Structure 2 as the predominant isomer. Its greater stability may be attributed to reduced steric repulsion between the nitro groups. In Structure 1, the NO_2_ substituents are positioned closer to one another, resulting in stronger steric interactions than in Structure 2.

### 2.3. Calibration Graph and Analytical Characteristics

A linear relationship between absorbance and the mass concentration of vanadium(V) was obtained up to 29.1 ng mL^−1^ (*R*^2^ = 0.9988, *n* = 9). The proposed procedure provides a sevenfold preconcentration, based on an aqueous sample volume of 14 mL and a final diluted SRP volume of 2.0 mL. The calibration equation was *A* = 32.1*γ* − 0.005, where *γ* is the vanadium(V) concentration (μg mL^−1^). The SDs of the slope and intercept were 0.4 and 0.006, respectively, indicating that the intercept is close to zero. 

The apparent molar absorptivity calculated from the slope of the calibration equation was 1.63 × 10^6^ L mol^−1^ cm^−1^. A similar value, 1.59 × 10^6^ L mol^−1^ cm^−1^, was obtained by averaging the calculated molar absorptivities for the eight non-zero experimental points. The limit of detection (LOD) and the limit of quantification (LOQ) were calculated as three and ten times the SD of the blank (*n* = 10), respectively, divided by the slope of the calibration graph. The resulting LOD and LOQ were 1.7 ng mL^−1^ and 5.7 ng mL^−1^, respectively.

The robustness of the proposed procedure was evaluated by introducing deliberate variations in key experimental parameters: (i) H_2_SO_4_ concentration (8.0 and 8.9 × 10^−3^ M), (ii) 3,5-DNC concentration (1.00 × 10^−4^ M and 1.14 × 10^−4^ M), (iii) TX-114 mass fraction (0.86% and 0.93%), and (iv) incubation temperature (65 and 70 °C). These minor variations had no significant impact on analytical performance, confirming the method’s reliability and robustness under standard laboratory operating conditions.

### 2.4. Effect of Foreign Ions

The effect of foreign ions on the determination of V(V) is summarized in Table 2. Large amounts of common ions, including alkali and alkaline earth metal ions, as well as NO_3_^−^, Cl^−^, and F^−^, did not interfere with the determination. Several metal ions, including Ni(II), Re(VII), Mn(II), Al(III), Zn(II), and Cu(II), were also tolerated at relatively high concentrations. Additionally, a 10-fold excess of Cd(II), 20-fold excesses of Co(II) and U(VI), and 50-fold excesses of Cr(III) and W(VI) did not affect the analysis.

The most significant interferences were caused by Mo(VI) and Fe(III). This limitation should be considered when selecting samples suitable for analysis, and appropriate sample pretreatment may be required for complex matrices containing elevated concentrations of these ions. If necessary, a 5-fold excess of Fe(III) can be effectively masked by the addition of F^−^. 

### 2.5. Application to Real Samples

The applicability of the developed method was evaluated by analyzing spent vanadium catalysts (SVCs) and vanadium-based dietary supplements.

The results obtained for the catalyst samples (Table 3) and dietary supplements (Table 4) were in good agreement with those obtained using two alternative methods [40,42]. To statistically evaluate the agreement between the results, an F-test was first performed to compare the variances, followed by a Student’s *t*-test to compare the mean values. The statistical analyses were performed at a 95% confidence level (α = 0.05), and differences were considered statistically significant at *p* < 0.05. For the dietary supplements, the experimentally determined vanadium contents were also compared with the manufacturer-declared values using the same statistical procedure. No statistically significant differences were observed between the vanadium contents determined by the proposed method and the manufacturer-declared values.

### 2.6. Comparison with Existing Spectrophotometric Methods for Vanadium Determination

Table 5 compares selected analytical characteristics of the method with those of previously reported UV–Vis spectrophotometric methods for vanadium determination. The proposed procedure combines operational simplicity, high sensitivity, and environmental friendliness. Its simplicity stems from the elimination of centrifugation and salting-out agents, both of which are commonly required in conventional CPE procedures. In addition, the reagent solutions are sufficiently stable (for at least 1 month) and do not require daily preparation, unlike those used in the previously reported pyrogallol methods [38,40].

The apparent molar absorptivity under sevenfold volume preconcentration conditions was approximately 1.6 × 10^6^ L mol^−1^ cm^−1^, which is substantially higher than the values reported for methods based on 3,5-DNC combined with other preconcentration techniques, including liquid–liquid extraction (LLE, ε = 1.7 × 10^5^ L mol^−1^ cm^−1^) [24] and solvent flotation (ε = 2.2 × 10^5^ L mol^−1^ cm^−1^) [25]. Moreover, the LOD and LOQ compare favorably with those reported for modern miniaturized techniques such as micro-CPE (MCPE) [51], dispersive liquid–liquid semi-microextraction (DLLSME) [52], and ion pair-based surfactant-assisted dispersive liquid–liquid microextraction with solidification of floating organic drop (IP-SA-DLLME-SFOD) [53].

Another advantage of the present procedure is the reduced amount of reagents required compared with existing methods. This is evident from the optimal mass fraction of the non-ionic surfactant relative to those used in other CPE procedures [38,42], as well as from the concentration of the chelating reagent (3,5-DNC) compared with those of 4-nitrocatechol [36] and pyrogallol [38,40].

To provide a more comprehensive evaluation of the proposed method, the principles of white analytical chemistry (WAC) were applied using the RGBfast model [54]. The proposed procedure was compared with four previously reported methods employing different preconcentration techniques: LLE [24], flotation [25], DLLSME [52], and IP-SA-DLLME-SFOD [53]. As shown in Figure 13, the proposed method achieved the highest score for the greenness component. It also performed well in the blueness and redness components but ranked slightly lower in overall performance (whiteness) than the IP-SA-DLLME-SFOD method reported by Mortada et al. [53], with scores of 60 and 61 points, respectively.

**Table 5:** Comparison with other spectrophotometric methods for V determination [40].

Reagent(s)	Preconcentration Technique	Extraction Solvent(s)	LOD, μg L^−1^	Linear Range, μg L^−1^	*λ*_max_, nm	pH	Sample	Ref.
3,5-DNC + BG	LLE	CCl_4_	NR	5–300	630	0.025 M H_2_SO_4_	Alums	[24]
3,5-DNC + RB	Flotation	Cyclohexane, hexane, and acetone	3	Up to 300	555	2.0–2.5	Vegetables	[25]
APANOL	–	–	NR	100–4000	533	3.0	Rice and flour	[55]
Br-PADAP	CPE	TX114	2.2	7–300	603	3.5	Water samples	[56]
CHMFC	LLE	CH_2_Cl_2_	55.9	Up to 1100	425	0.2 M CH_3_COOH	Synthetic and technical samples	[57]
CV + KI	–	–	625	100–10,200	588	4.0	Water, soil, steel, biological and pharmaceutical samples	[58]
DHMPhB	DLLSME	Chloroform + methanol	3.9 (V^IV,V^) 11.4 (V^V^)	13–612 (V^IV,V^) 38–1200 (V^V^)	520 525	4.0	Tap water and synthetic mixtures	[52]
EDTA + Safranin T	UA-CPE	TX114	0.53 (V^V^) 0.26 (V^IV^)	2–180 (V^V^) 1–40 (V^IV^)	530	4.0 (V^V^) 5.0 (V^IV^)	Vegetal oils and vinegar	[59]
GB + KBrO_3_	–	–	0.31	1–100	537	2.0	Water samples	[60]
HTAR + TTC	LLE	Chloroform	4.6	15–2000	549	4.7	Catalysts and pharmaceuticals	[61]
ICQ	LLE	Chloroform	210	Up to 7000	407	2 M CH_3_COOH	Synthetic mixtures	[62]
*N*-BPHA	LLE	Chloroform	NR	Up to 1500	530	≥2.4 M HCl	Natural waters	[63]
NC + CPC	MM-CL-CPE	TX114	0.6	2–305	670	5.5	Mineral water, catalysts, and dietary supplement	[36]
PAR	MCPE	TX114	5.51	50–600	568	5.5	Tap water	[51]
PG + A336	IL-MM-CL-CPE	TX114	0.11	Up to 3.6	309	3.4	Water, dietary supplements, and catalysts	[40]
PG + Safranin T	UA-CPE	TX114	0.58	2–500	533	6.0	Beverage samples	[38]
TA + CTAB	IP-SA-DLLME-SFOD	1-Undecanol	1.8	6–1000	600	6.0	Fruit juice samples	[53]
TAN + H_2_O_2_	MWA-CL-CPE	TX100	1.4	Up to 760	607	5 × 10^−3^ M H_2_SO_4_	Water, Al-alloy, catalyst, and solution for infusion	[42]
3,5-DNC + BAC	MM-CL-CPE	TX114	1.7	Up to 29.1	426	(8–9) × 10^−3^ M H_2_SO_4_	Dietary supplements and spent vanadium catalysts	This work

Abbreviations: 3,5-DNC, 3,5-dinitrocatechol; A336, Aliquat 336; APANOL, 1-[(4-antipylazo)]-2-naphthol; BAC, benzalkonium chloride; BG, brilliant green; Br-PADAP, 2-(5-bromo-2-pyridylazo)-5-diethylaminophenol; CHMFC, 6-chloro-3-hydroxy-7-methyl-2-(2′-furyl)-4H chromen-4-one; CL-CPE, centrifuge-less cloud point extraction; CPC, cetylpyridinium chloride; CPE, cloud point extraction; CTAB, cetyltrimethylammonium bromide; CV, crystal violet; DHMPhB, 6,7-dihydroxybenzopyrylium bromide; DLLSME, dispersive liquid–liquid semi-microextraction; GB, Gallamine blue; HTAR, 6-hexyl-4-(2-thiazolylazo)-resorcinol; ICQ, iodochlorhydroxyquin; IP-SA-DLLME-SFOD, ion pair-based surfactant-assisted dispersive liquid–liquid microextraction with solidification of floating organic drop; LLE, liquid–liquid extraction; MCPE, micro-CPE; MWA-CL-CPE, microwave-assisted centrifuge-less CPE; MM-CL-CPE, mixed micelle mediated centrifuge-less CPE; *N*-BPHA, *N*-benzoyl-*N*-phenylhydroxylamine; NC, 4-nitrocatechol; NR, not reported; PAR, 4-(2-pyridylazo)resorcinol; PG, pyrogallol; RB, Rhodamine B; TA, tannic acid; TAN, 1-(2-thiazolylazo)-2-naphthol; TTC, 2,3,5-thriphenyl-2*H*-tetrazolium chloride; TX100, Triton X-100; TX114, Triton X-114; UA-CPE, ultrasonic-assisted CPE.

### 2.7. Evaluation of the Proposed Method Using Other Metrics

In addition to the comparative RGBfast model, the proposed method was evaluated using several widely applied analytical metrics, namely the Analytical GREEnness Metric (AGREE) [64], the Sample Preparation Metric for Sustainability (SPMS) [65], and the Blue Applicability Grade Index (BAGI) [66]. The evaluation results are presented in Figure 14.

AGREE is one of the most widely used tools for assessing the greenness of analytical methods. It is based on the 12 principles of Green Analytical Chemistry (GAC). The proposed method achieved an AGREE score of 0.56 (Figure 14a), which is above the reference value of 0.50. The corresponding pie chart contains several green and yellow-green sectors, reflecting favorable performance with respect to GAC principles 4, 6, 8, 9, 11, and 12.

The SPMS and BAGI scores were 5.37 (Figure 14b) and 60.0 (Figure 14c), respectively, both exceeding their corresponding benchmark values of 5.00 and 50.0. According to the SPMS and BAGI criteria, the proposed method demonstrates an acceptable level of sustainability and practical applicability, respectively.

Despite the relatively favorable scores obtained using these evaluation tools, several drawbacks of the proposed method should also be noted, primarily related to the need for prolonged heating (60 min) followed by a cooling step. The lower repeatability of the method, compared with other CPE–spectrophotometric methods recently developed in our laboratory [36,40], may be related to the possible contribution of the second isomeric form (Structure 1), which has different spectrophotometric characteristics.

## 3. Materials and Methods

### 3.1. Reagents and Solutions

All reagents were purchased from Merck (Schnelldorf, Germany). A vanadium(V) stock solution (2.0 × 10^−4^ M) was prepared from ammonium metavanadate (NH_4_VO_3_, puriss. p.a.). Working V(V) solutions (1.0 × 10^−5^ M) were prepared by appropriate dilution. A 3,5-DNC (≥98%) solution (8.0 × 10^−4^ M) was prepared in 20% (*v*/*v*) ethanol, as described in Refs. [23,24]. An aqueous BAC (≥95%) solution was prepared at a concentration of 1.0 × 10^−2^ M. TX114 (laboratory grade) was used as a 6.3% (*w*/*w*) aqueous solution. The sulfuric acid solution had a concentration of 2.5 × 10^−1^ M.

All solutions were prepared using deionized water (18.2 MΩ cm; ELGA-Veolia LabWater, High Wycombe, UK).

### 3.2. Instrumentation

Absorbance measurements were carried out using Ultrospec 3300 Pro and Spectronic Camspec M550 spectrophotometers (Garforth, UK), both of which were equipped with 10 mm path-length semimicro cuvettes. CPE experiments were conducted in a GFL 1023 thermostatic water bath (LAUDA-GFL Gesellschaft für Labortechnik mbH, Burgwedel, Germany), and pH was measured using a WTW InoLab 7110 pH meter (WTW-Xylem Analytics, Weilheim in Oberbayern, Germany).

### 3.3. Sample Preparation

SVCs used for SO_2_ oxidation were supplied by Holding KCM 2000 (Bulgaria). Three vanadium-containing dietary supplements were purchased from a local pharmacy: (1) Chromium & Vanadium (Natural Factors^®^, Coquitlam, BC, Canada), (2) Vanadyl Sulfate (Source Naturals^®^, Scotts Valley, CA, USA), and (3) Vanadium, Vanadyl Sulfate with Chromium (Nutricost^®^, Vineyard, UT, USA). According to the manufacturers’ specifications, each Chromium & Vanadium capsule contains 25 μg of vanadium (as citrate), whereas the other two products contain 2 mg of vanadium per dosage unit (capsule or tablet) in the form of VOSO_4_.

Sample preparation was performed according to the procedures previously reported: the SVC samples were prepared according to the description provided in Ref. [61], while the dietary supplements were prepared following the procedure reported in Ref. [36].

### 3.4. Optimization Procedure

Appropriate volumes of TX-114, V(V), 3,5-DNC, H_2_SO_4_, and BAC solutions were transferred into 15 mL conical-bottom graduated centrifuge tubes. The mixtures were diluted with water to achieve a final volume of 14 mL and placed in a thermostatic water bath at 50–70 °C for 20–100 min. The tubes were briefly chilled under running water and subsequently kept in a freezer at −20 °C for 25 min to facilitate phase separation. The SRPs were isolated by decanting the upper aqueous phases. Subsequently, 300 μL of ethanol was added to each SRP. The mixtures were diluted with water to a final volume of 2.0 mL and shaken until complete homogenization. Finally, the resulting solutions were transferred to semimicro cuvettes to measure absorbance.

### 3.5. Recommended Analytical Procedure

A 2.0 mL aliquot of 6.3% TX-114 solution was transferred into a 15 mL graduated centrifuge tube. Subsequently, an aliquot of the analyzed solution containing 80–407 ng V(V), 1.8 mL of 8.0 × 10^−4^ M 3,5-DNC solution, 0.5 mL of 2.5 × 10^−1^ M H_2_SO_4_ solution, and 1.3 mL of 1.0 × 10^−2^ M BAC solution was added. The mixture was diluted with water to a final volume of 14 mL and thoroughly mixed. The resulting solution corresponded to final V(V) concentrations of 5.7–29.1 ng mL^−1^. The pH of this solution should be 2.0–2.1; if necessary, the volume of the H_2_SO_4_ solution added should be adjusted accordingly.

The tube was incubated in a thermostatic water bath at 65 °C for 60 min. It was then cooled under running water and subsequently placed in a freezer at −20 °C for 25 min to facilitate phase separation. The upper aqueous phase was removed by decantation, leaving the viscous SRP. Subsequently, 300 μL of ethanol was added to the SRP. The mixture was diluted with water to a final volume of 2.0 mL and shaken until complete homogenization. Finally, the resulting solution was transferred to a semimicro cuvette, and the absorbance was measured at 426 nm against a reagent blank prepared under identical conditions. The V(V) concentration was determined from the previously constructed calibration curve.

### 3.6. Theoretical Section

The ground-state equilibrium geometries of the two isomeric forms of the anionic component of the extracted complex were optimized at the B3LYP/6-311G level of theory. The charge and spin multiplicity were set to −1 and 1, respectively. Frequency calculations were subsequently performed to confirm that the optimized structures corresponded to true minima on the potential energy surface by verifying the absence of imaginary frequencies. Vertical excitation energies were then calculated to simulate the electronic absorption spectra and compare them with the experimental spectrum. All calculations were carried out using GAUSSIAN 16 [67], and structural visualization was performed with ChemCraft version 1.8 [68].

## 4. Conclusions

A novel mixed-micelle-mediated, centrifuge-less cloud point extraction (MM-CL-CPE)–spectrophotometric method was developed for the determination of vanadium(V). The proposed procedure combines operational simplicity, high sensitivity, and environmental friendliness. Its analytical performance was demonstrated through the successful analysis of real samples, while its whiteness and sustainability were comprehensively assessed using modern metric tools, namely RGBfast, AGREE, SPMS, and BAGI.

## Figures and Tables

**Figure 1:**
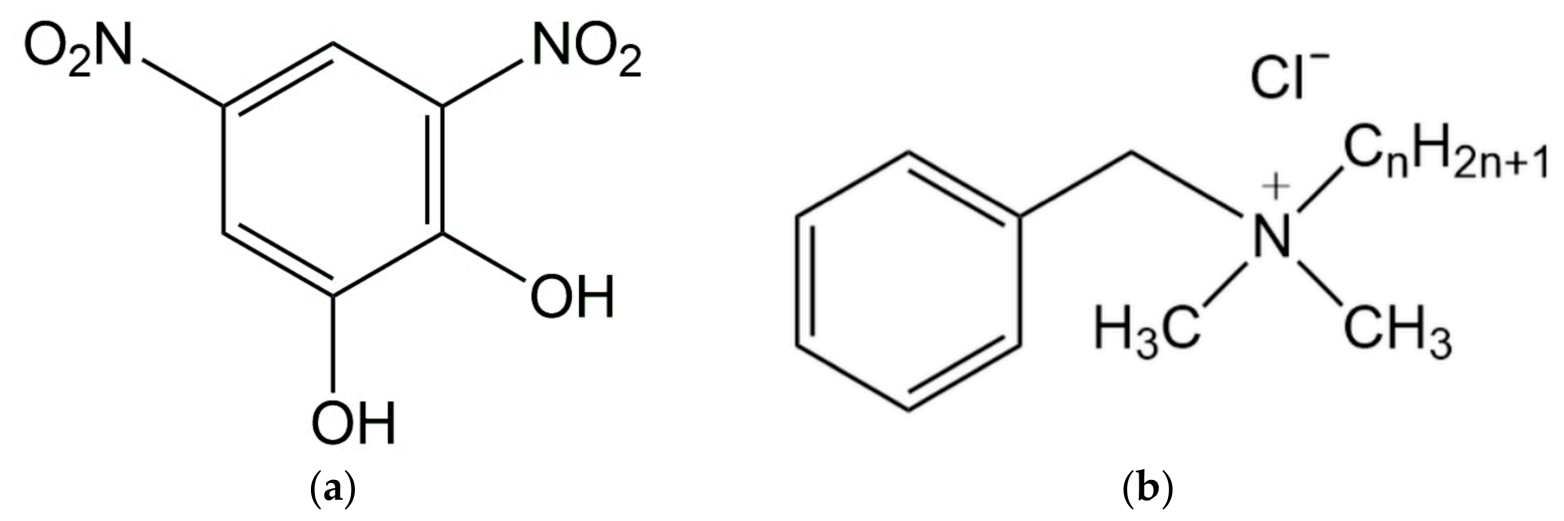
Structural formulae of 3,5-Dinitrocatechol (3,5-DNC; 3,5-dinitrobenzene-1,2-diol) (**a**) and benzalkonium chloride (BAC; n = 8, 10, 12, 14, 16, and 18) (**b**).

**Figure 2:**
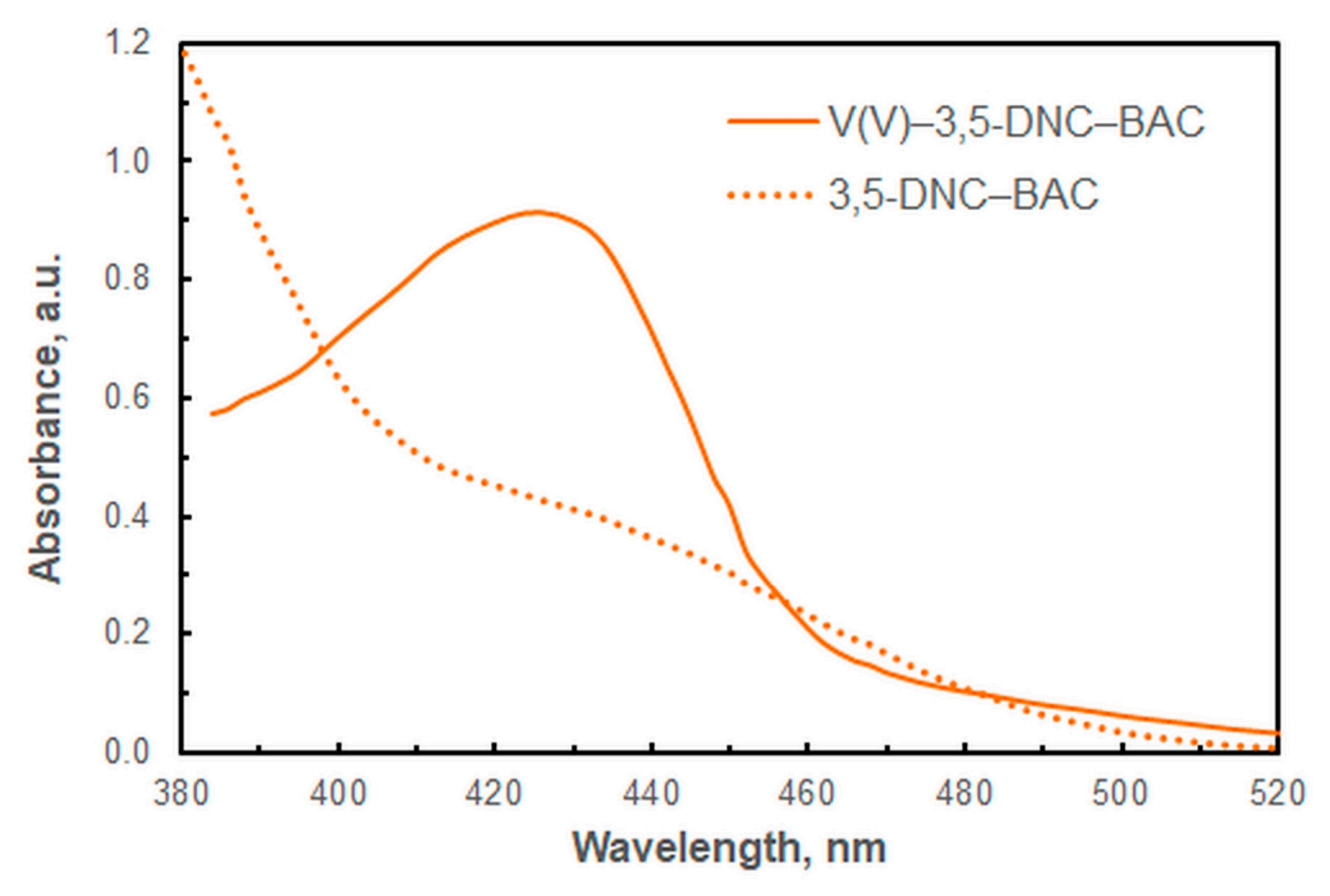
Spectra of the V(V)–3,5-DNC–BA^+^ complex against blank (1) and the blank against water (2): *c*_V_ = 5.7 × 10^−7^ M, *c*_3,5-DNC_ = 1.0 × 10^−4^ M, *c*_BAC_ = 9.3 × 10^−4^ M, *c*_H_2_SO_4__ = 8.9 × 10^−3^ M, *w*_TX114_ = 0.9%, *t*_inc_ = 60 min at 65 °C, *V*_SRP_ = 2.0 mL.

**Figure 3:**
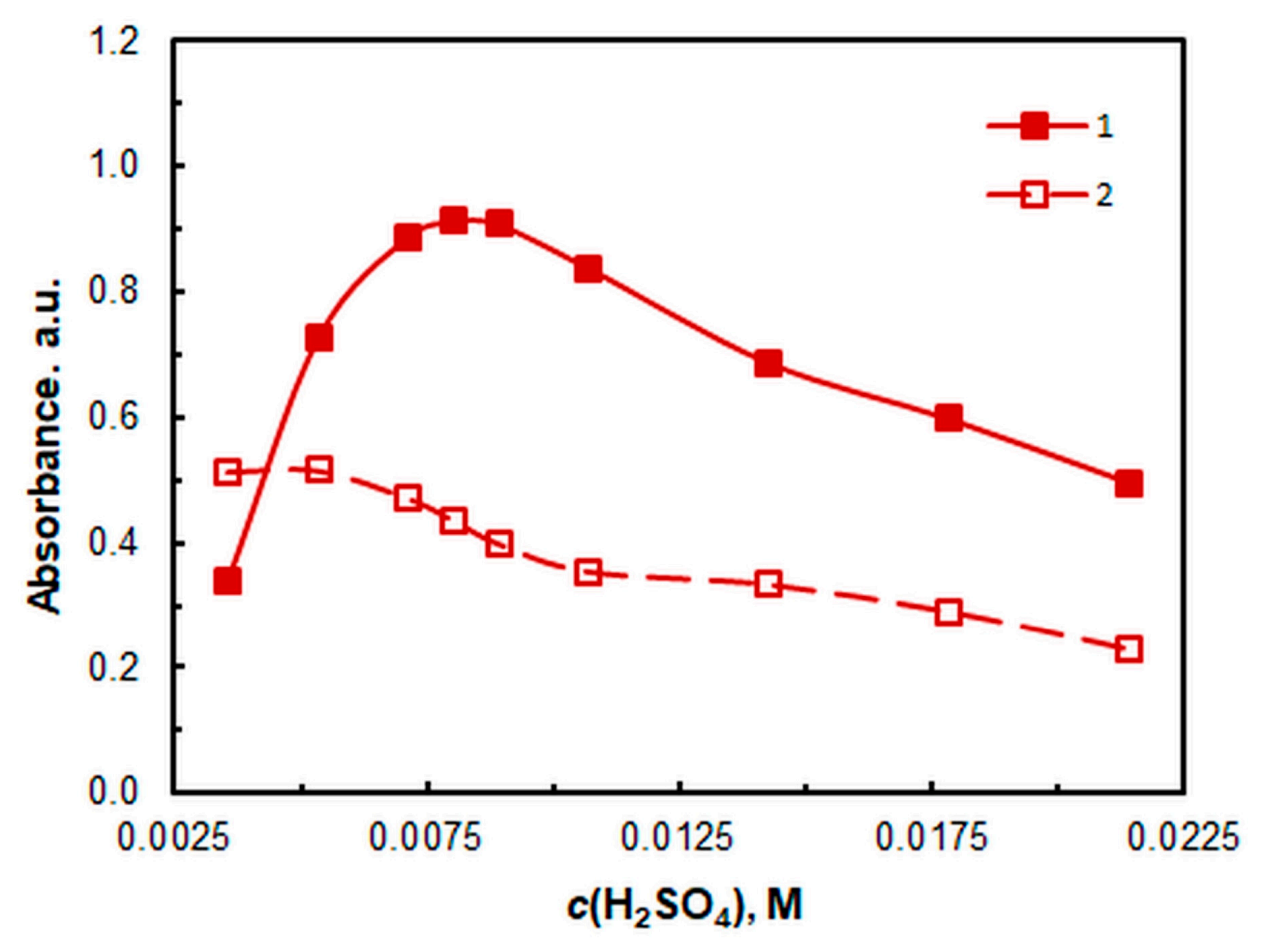
Effect of H_2_SO_4_ concentration. *c*_V_ = 5.7 × 10^−7^ M, *c*_3,5-DNC_ = 1.0 × 10^−4^ M, *c*_BAC_ = 9.3 × 10^−4^ M, *w*_TX114_ = 0.9%, *t*_inc_ = 60 min at 65 °C, *V*_SRP_ = 2.0 mL, *λ* = 426 nm.

**Figure 4:**
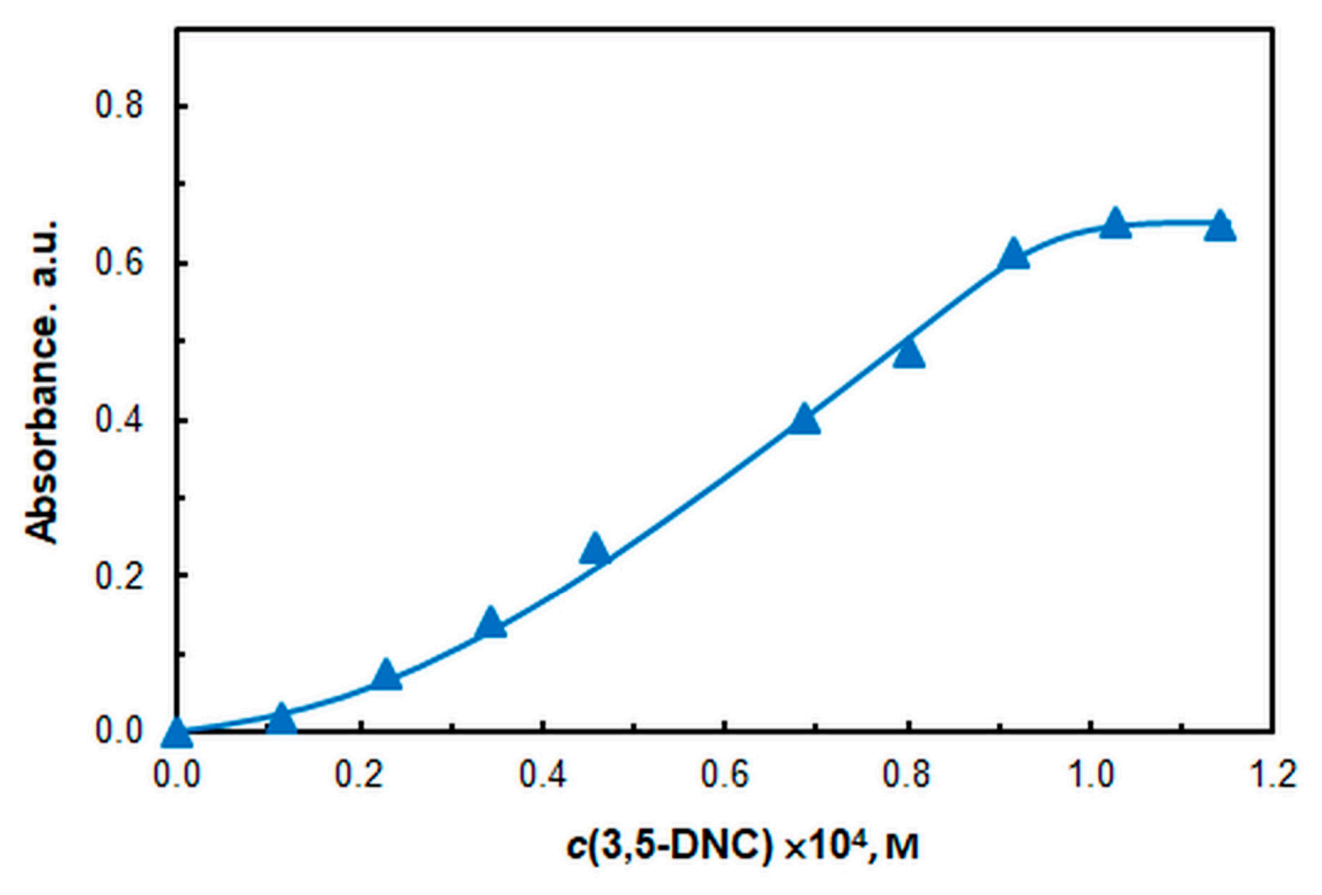
Effect of 3,5-DNC concentration. *c*_V_ = 4.1 × 10^−7^ M, *c*_BAC_ = 9.3 × 10^−4^ M, *c*_H_2_SO_4__ = 8.9 × 10^−3^ M, *w*_TX114_ = 0.9%, *t*_inc_ = 60 min at 65 °C, *V*_SRP_ = 2.0 mL, *λ* = 426 nm.

**Figure 5:**
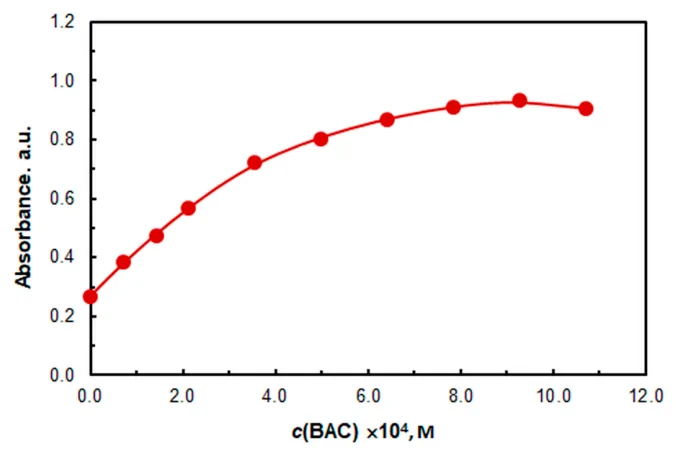
Effect of BAC concentration. *c*_V_ = 5.7 × 10^−7^ M, *c*_3,5-DNC_ = 1.0 × 10^−4^ M, *c*_H_2_SO_4__ = 8.9 × 10^−3^ M, *w*_TX114_ = 0.9%, *t*_inc_ = 60 min at 65 °C, *V*_SRP_ = 2.0 mL, *λ* = 426 nm.

**Figure 6:**
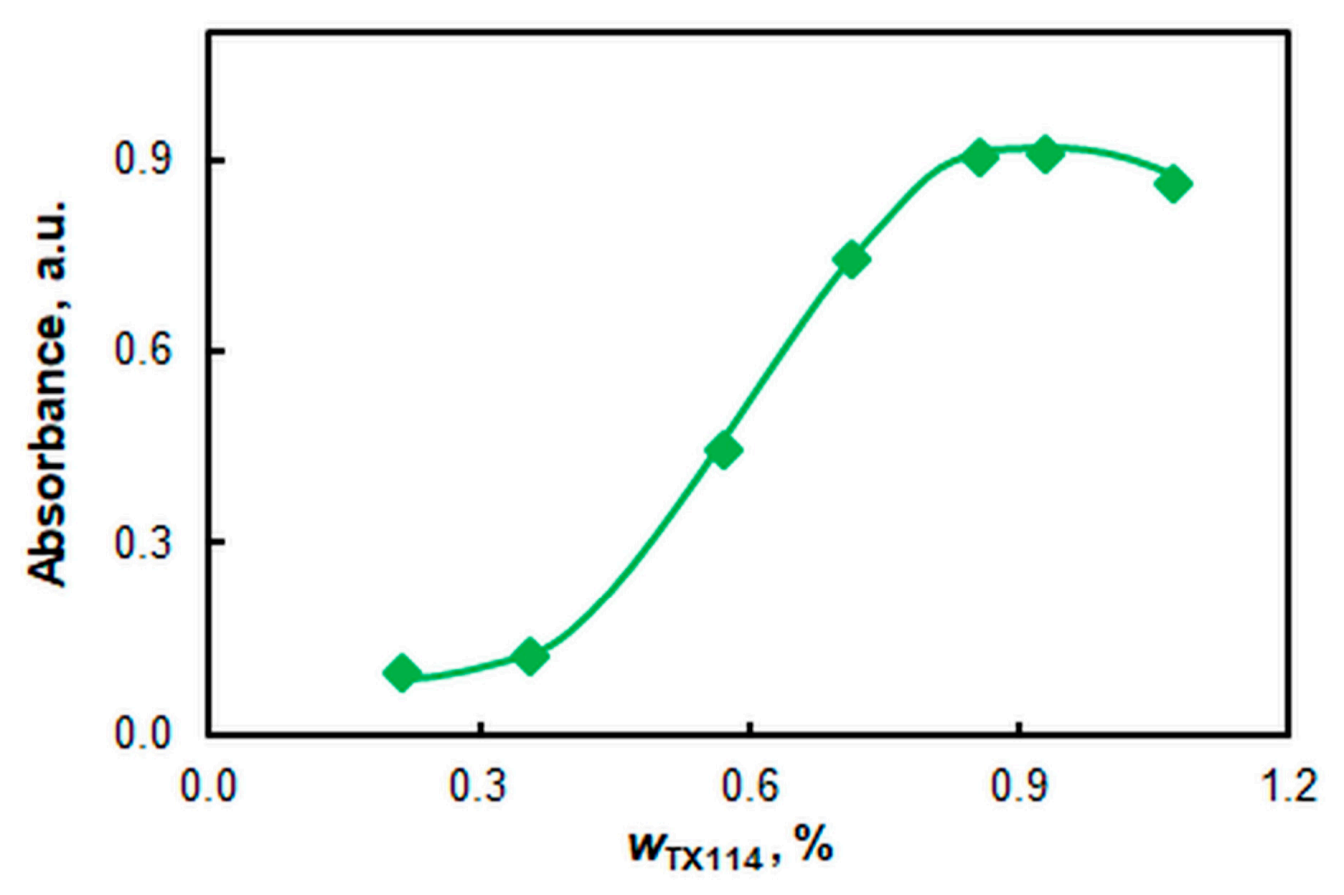
Effect of TX114 mass fraction. *c*_V_ = 5.7 × 10^−7^ M, *c*_3,5-DNC_ = 1.0 × 10^−4^ M, *c*_BAC_ = 9.3 × 10^−4^ M, *c*_H_2_SO_4__ = 8.9 × 10^−3^ M, *t*_inc_ = 60 min at 65 °C, *V*_SRP_ = 2.0 mL, *λ* = 426 nm.

**Figure 7:**
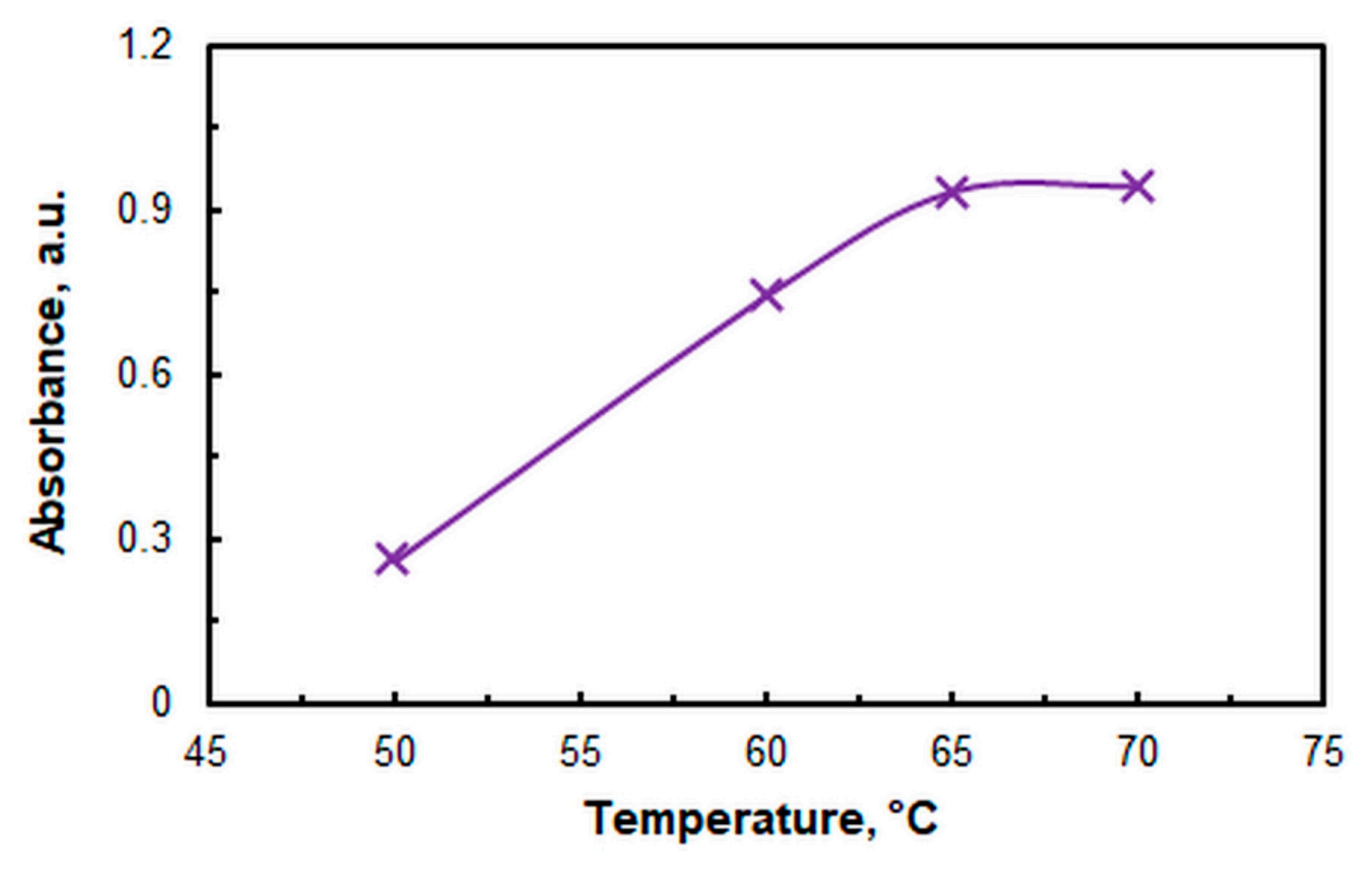
Effect of incubation temperature. *c*_V_ = 5.7 × 10^−7^ M, *c*_3,5-DNC_ = 1.0 × 10^−4^ M, *c*_BAC_ = 9.3 × 10^−4^ M, *c*_H_2_SO_4__ = 8.9 × 10^−3^ M, *w*_TX114_ = 0.9%, incubation time of 90 min, *V*_SRP_ = 2.0 mL, *λ* = 426 nm.

**Figure 8:**
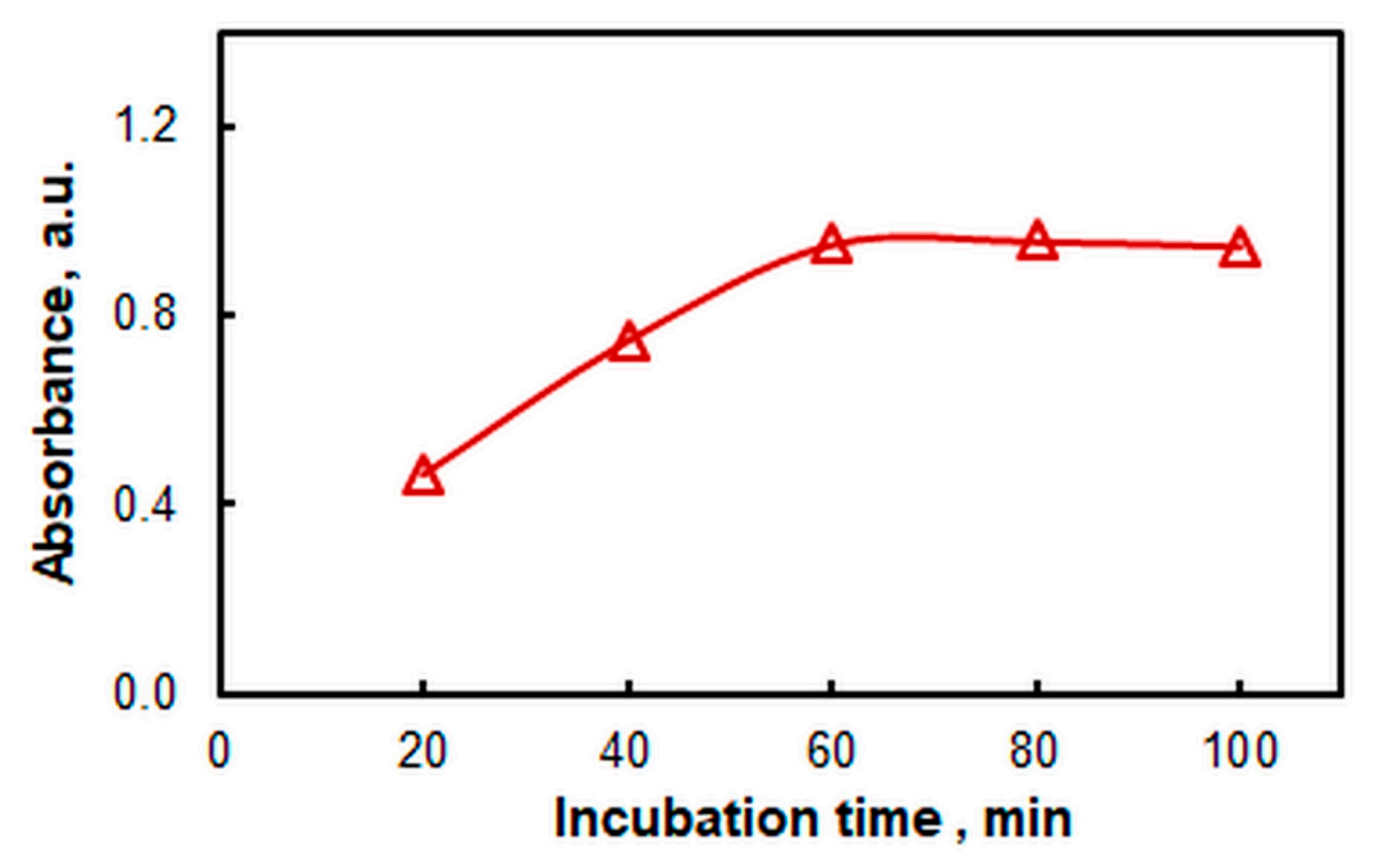
Effect of incubation time. *c*_V_ = 5.7 × 10^−7^ M, *c*_3,5-DNC_ = 1.0 × 10^−4^ M, *c*_BAC_ = 9.3 × 10^−4^ M, *c*_H_2_SO_4__ = 8.9 × 10^−3^ M, *w*_TX114_ = 0.9%, incubation temperature of 65 °C, *V*_SRP_ = 2.0 mL, *λ* = 426 nm.

**Figure 9:**
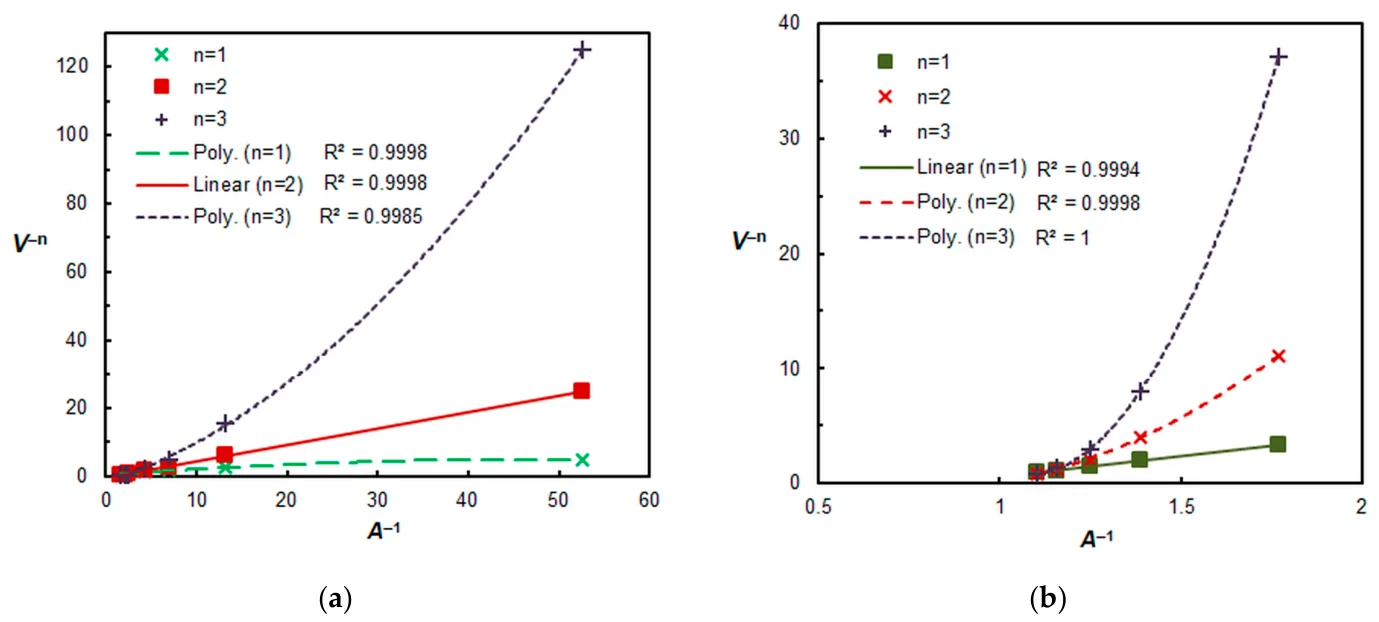
Determination of the 3,5-DNC:V(V) (**a**) and BAC:V(V) (**b**) molar ratios using the Asmus method.

**Figure 10:**
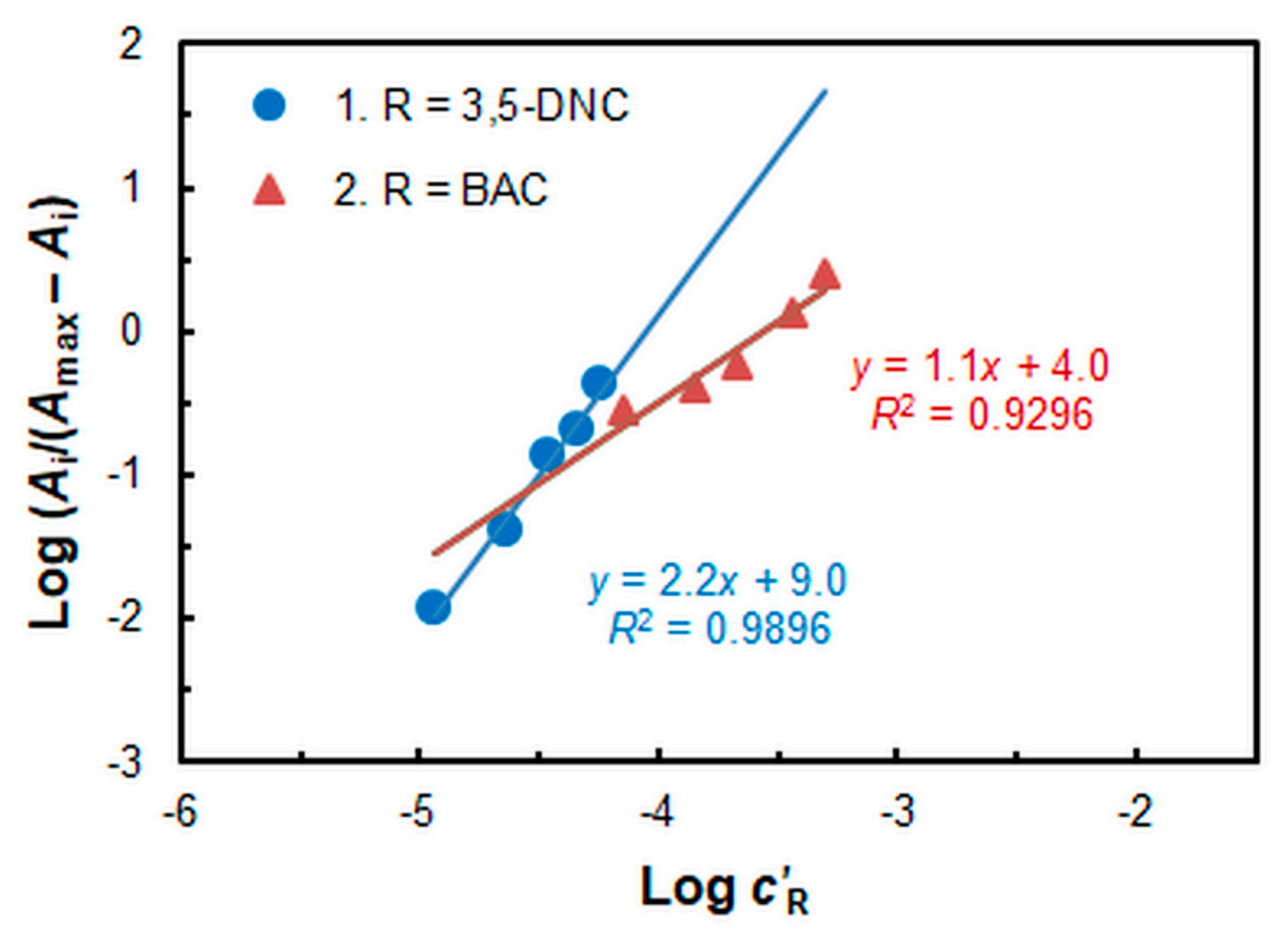
Determination of the 3,5-DNC:V(V) (straight line 1) and BAC:V(V) (straight line 2) molar ratios using the mobile equilibrium method.

**Figure 11:**
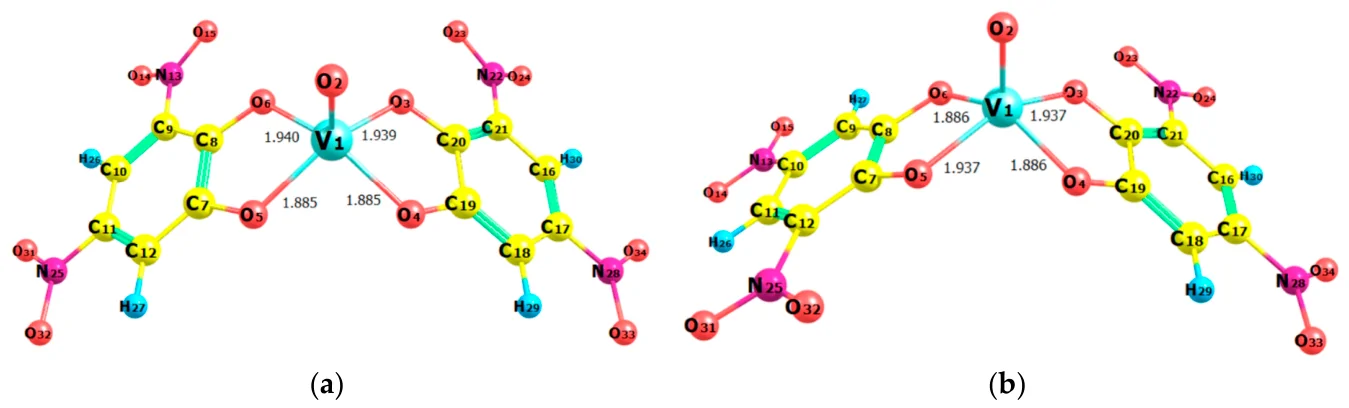
Optimized ground-state equilibrium geometries of the two isomers: syn (Structure 1) (**a**) and anti (Structure 2) (**b**).

**Figure 12:**
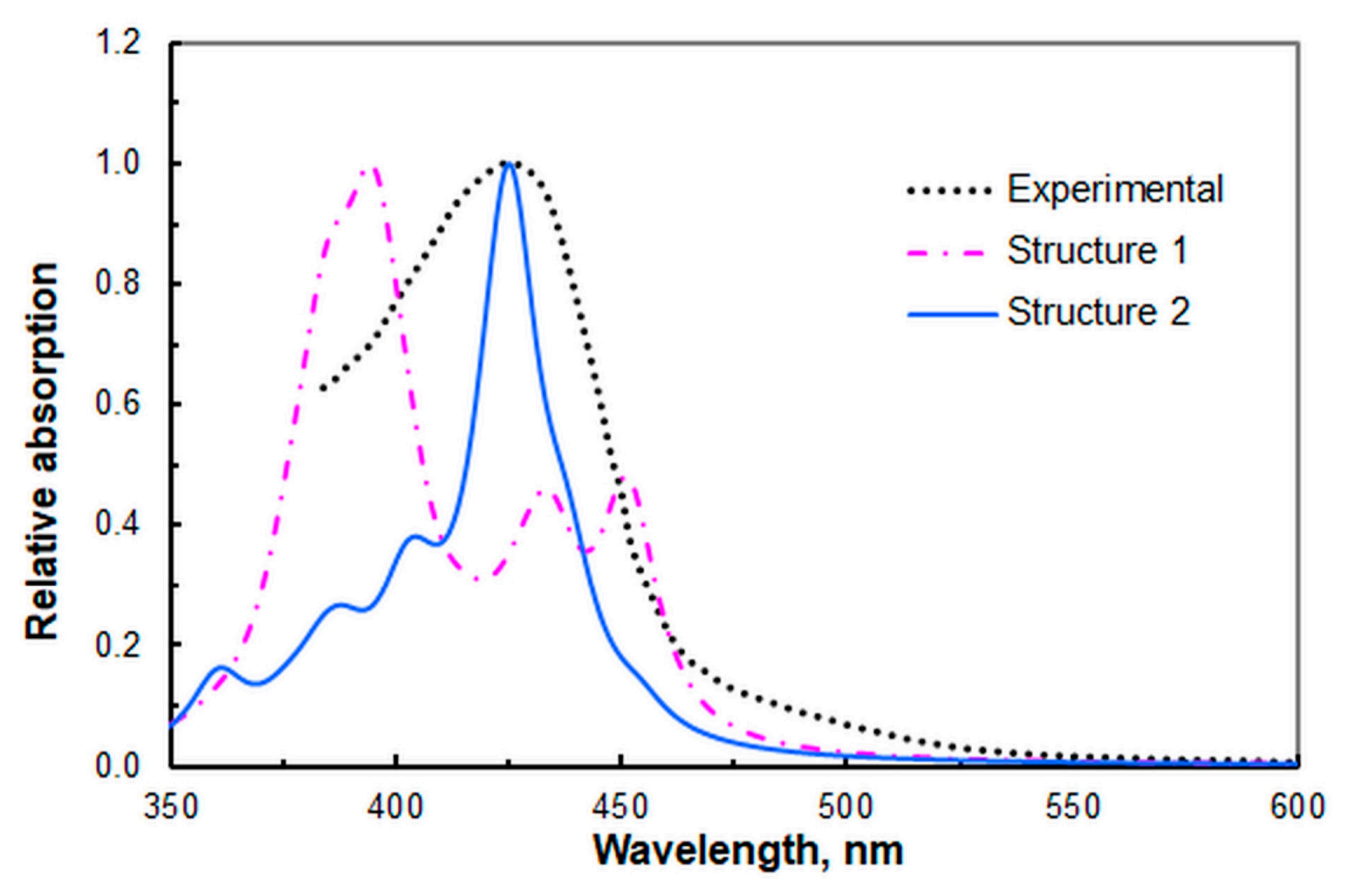
Comparison of the normalized experimental and simulated absorption spectra. The theoretical spectra were generated using Lorentzian band broadening and a scaling factor of 1.015.

**Figure 13:**
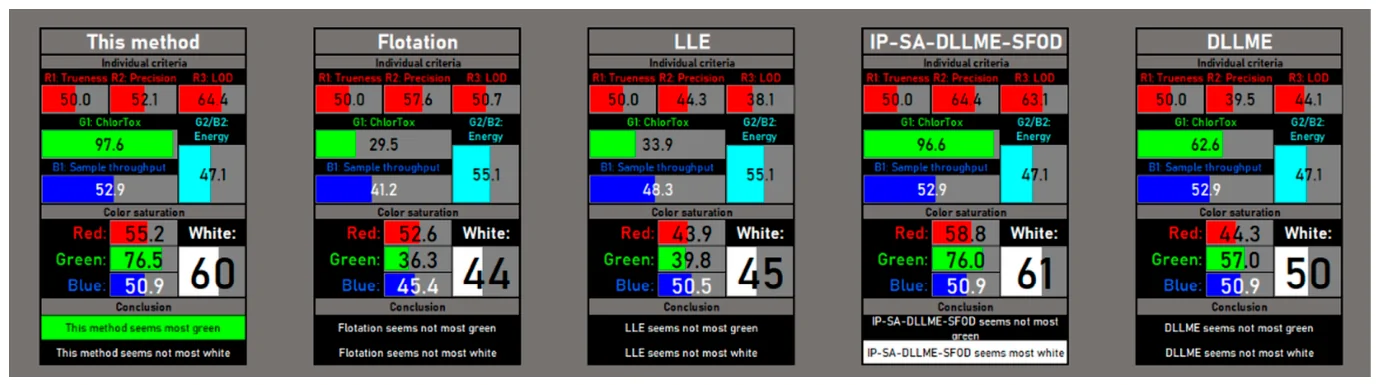
Comparison of the proposed method with other spectrophotometric methods for vanadium determination using the RGBfast model.

**Figure 14:**
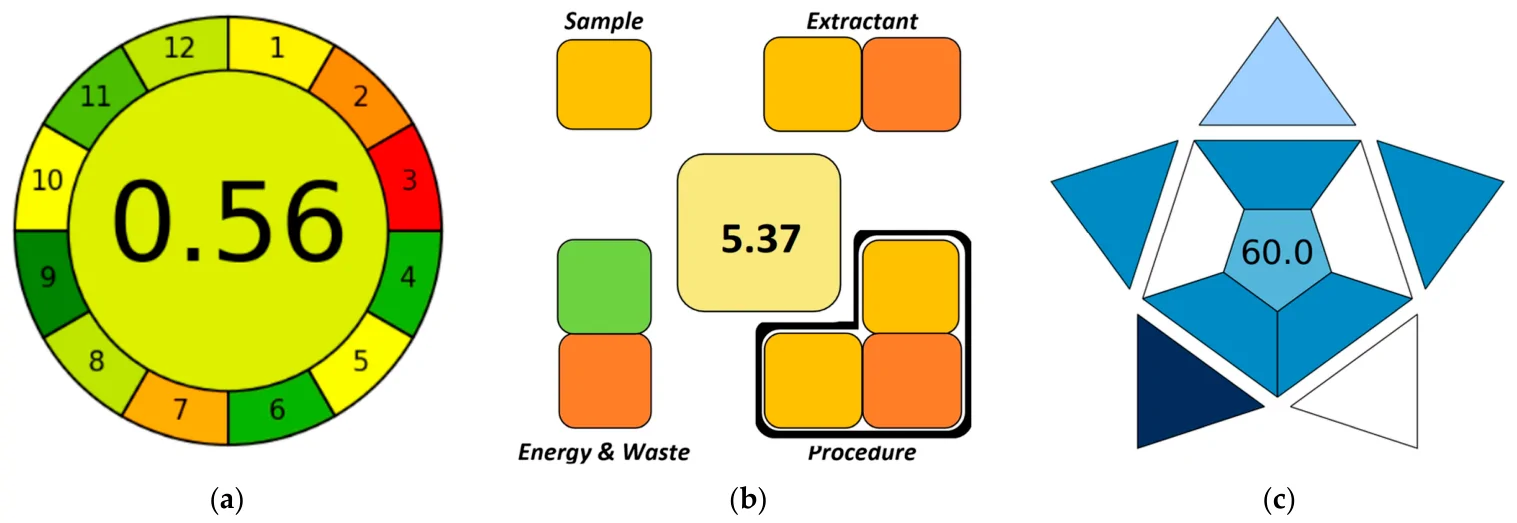
Evaluation of the proposed method using AGREE (**a**), SPMS (**b**), and BAGI (**c**).

**Table 1:** Optimized experimental parameters.

Parameter	Optimum
Wavelength, nm	426
Concentration of H_2_SO_4_, M	(8–9) × 10^−3^
Concentration of 3,5-DNC, M	1.0 × 10^−4^
Concentration of BAC, M	9.3 × 10^−4^
Mass fraction of TX114, %	0.9
Incubation time, min	60
Incubation temperature, °C	65
Chilling time (at −20 °C), min	25
Volume of the sample *, mL	14.0
Volume of the final (diluted) SRP, mL **	2.0

* Experiments performed in 15 mL test tubes. ** Diluted to the 2.0 mL mark with 300 μL of ethanol + water.

**Table 2:** The effect of foreign ions on the determination of V(V).

Ion	Added Salt Formula	Ion:V(V) Mass Ratio	*R*, %
Al(III)	Al_2_(SO_4_)_3_·18H_2_O	500	98.6
Ca(II)	Ca(NO_3_)_2_	2000 *	105
Cd(II)	Cd(NO_3_)_2_·4H_2_O	10	98.8
		20	106
Cl^−^	NaCl	2000 *	95.6
Co(II)	CoSO_4_·7H_2_O	20	98.2
Cr(III)	Cr_2_(SO_4_)_3_	50	101
Cr(VI)	K_2_CrO_4_	5	106
Cu(II)	Cu(NO_3_)_2_·3H_2_O	200	101
F^−^	NaF	2000 *	103
Fe(III)	Fe_2_(SO_4_)_3_	5	114
		5 **	102
I^−^	KI	2000 *	95.0
K^+^	K_2_SO_4_	2000 *	95.8
Li^+^	Li_2_SO_4_·H_2_O	2000 *	105
Mn(II)	MnSO_4_·5H_2_O	750	103
Mo(VI)	Na_2_MoO_4_	1	102
		2	108
		5	122
Ni(II)	NiSO_4_·7H_2_O	1000	101
Re(VII)	NH_4_ReO_4_	1000	98.7
Sr(II)	Sr(NO_3_)_2_	2000 *	98.7
Tartrate^2−^	KNaC_4_H_4_O_6_·4H_2_O	500	99.2
U(VI)	UO_2_(CH_3_CO_2_)_2_·H_2_O	20	98.6
W(VI)	Na_2_WO_4_·2H_2_O	50	102
Zn(II)	ZnSO_4_·7H_2_O	2000 *	98.4

* Higher mass ratios were not studied. ** Masked with 0.6 mg F^−^.

**Table 3:** Determination of V in spent vanadium catalysts (SVCs).

SVC Sample *	Vanadium Found **, %			RSD, %		
	Present Method	Alternative Method [42]	Alternative Method [40]	Present Method	Alternative Method [42]	Alternative Method [40]
Sample 1	2.81 ± 0.07	2.87 ± 0.11	2.82 ± 0.06	2.5	3.8	2.1
Sample 2	2.38 ± 0.08	2.32 ± 0.10	2.35 ± 0.04	3.4	4.3	1.7
Sample 3	2.13 ± 0.08	2.07 ± 0.08	–	3.8	4.1	–

* Supplied from Holding KCM 2000, Bulgaria. ** Mean ± SD, *n* = 4.

**Table 4:** Determination ^a^ of vanadium in vanadium-based dietary supplements.

Supplement	Manufacturer’s Declared Value Per Capsule or Tablet	Vanadium Found, μg		
		Present Method	Alternative Method [42]	Alternative Method [40]
Sample 1 ^b^	25 μg	24.3 ± 0.7	24.3 ± 0.9	24.5 ± 0.6
Sample 2 ^c^	2 mg	1984 ± 66	2043 ± 81	–
Sample 3 ^d^	2 mg	2049 ± 62	1977 ± 72	2024 ± 51

^a^ Mean ± SD, *n* = 4. ^b^ Natural Factors^®^, Canada, Chromium & Vanadium. ^c^ Source Naturals^®^, United States, Vanadyl Sulfate. ^d^ Nutricost^®^, United States, Vanadium (Vanadyl Sulfate with Chromium).

## Data Availability

Data are contained within the article.
